# Digital Care Programs for Chronic Hip Pain: A Prospective Longitudinal Cohort Study

**DOI:** 10.3390/healthcare10081595

**Published:** 2022-08-22

**Authors:** Dora Janela, Fabíola Costa, Anabela C. Areias, Maria Molinos, Robert G. Moulder, Jorge Lains, Virgílio Bento, Justin K. Scheer, Vijay Yanamadala, Steven P. Cohen, Fernando Dias Correia

**Affiliations:** 1 Sword Health Inc., Draper, UT 84043, USA; 2 Institute for Cognitive Science, University of Colorado Boulder, Boulder, CO 80309, USA; 3 Rovisco Pais Medical and Rehabilitation Centre, 3064-908 Tocha, Portugal; 4 Faculty of Medicine, Coimbra University, 3004-504 Coimbra, Portugal; 5 Department of Neurological Surgery, University of California, San Francisco, CA 94143, USA; 6 Department of Surgery, Frank H. Netter School of Medicine, Quinnipiac University, Hamden, CT 06473, USA; 7 Department of Neurosurgery, Hartford Healthcare Medical Group, Westport, CT 06103, USA; 8 Departments of Anesthesiology & Critical Care Medicine, Physical Medicine and Rehabilitation, Neurology, and Psychiatry and Behavioral Sciences, Johns Hopkins School of Medicine, Baltimore, MD 21287, USA; 9 Department of Anesthesiology and Physical Medicine and Rehabilitation and Anesthesiology, Uniformed Services University of the Health Sciences, Bethesda, MD 20814, USA; 10 Neurology Department, Centro Hospitalar e Universitário do Porto, 4099-001 Porto, Portugal

**Keywords:** musculoskeletal pain, physical therapy, telerehabilitation, digital therapy, eHealth, motion trackers

## Abstract

Chronic hip pain is a cause of disability worldwide. Digital interventions (DI) may promote access while providing proper management. This single-arm interventional study assesses the clinical outcomes and engagement of a completely remote multimodal DI in patients with chronic hip pain. This home-based DI consisted of exercise (with real-time biofeedback), education, and cognitive-behavioral therapy. Outcomes were calculated between baseline and program end, using latent growth curve analysis. Primary outcome was the Hip Disability and Osteoarthritis Outcome Score (HOOS). Secondary outcomes were pain, intent to undergo surgery, mental health, productivity, patient engagement (exercise sessions frequency), and satisfaction. Treatment response was assessed using a 30% pain change cut-off. A completion rate of 74.2% (396/534), alongside high patient engagement (2.9 exercise sessions/week, SD 1.1) and satisfaction (8.7/10, SD 1.6) were observed. Significant improvements were observed across all HOOS sub-scales (14.7–26.8%, *p* < 0.05), with 66.8% treatment responders considering pain. Marked improvements were observed in surgery intent (70.1%), mental health (54%), and productivity impairment (60.5%) (all *p* < 0.001). The high engagement and satisfaction reported after this DI, alongside the clinical outcome improvement, support the potential of remote care in the management of chronic hip conditions.

## 1. Introduction

Hip pain is a common source of disability that increases with age, affecting one in five adults over 65 years old [1]. Over 18 million individuals report chronic hip symptoms in the United States (US) [2], which are associated with reduced functionality and quality of life, as well as with mental distress [3,4,5]. The weak correlation between abnormal structural findings and hip pain and/or disability prompted a shift towards a biopsychosocial model in the management of these conditions [6,7]. Conservative treatment is prioritized over invasive procedures, including surgery [8,9,10,11,12]. However, surgery is still a common and increasing practice (even at younger ages [13]), with over one million total hip replacement procedures performed each year in the US [14]. Adding to higher risks of complications [15], the small differences in hip outcomes observed between surgery and conservative treatments raise questions on the cost-effectiveness of surgical solutions [16,17,18].

Among conservative treatments, exercise and education combined with other approaches targeting biopsychosocial factors have been reported as the most effective [19,20]. There is moderate evidence that exercise interventions reduce pain by 19–28% and improve functionality by 11–24% in individuals with hip osteoarthritis [19,20], alongside improvements in depression [19,20]. Scientific research on productivity domains is still scarce, nevertheless, preliminary findings indicate improvements in absenteeism with self-management programs [21].

However, the success of such interventions depends largely on adherence, a well-known predictor of better outcomes among people with musculoskeletal (MSK) conditions [22], namely hip osteoarthritis [23]. Treatment adherence is dependent on a number of factors, including access to practitioners and facilities, time, disability, mental health, and perceived effectiveness of conservative treatment [24,25]. Digital modalities have been reported to result in similar outcomes to in-person interventions for hip conditions [26], namely on post-surgical rehabilitation [27,28,29,30] and as a conservative approach for hip osteoarthritis [26,31,32,33,34,35], with other chronic hip conditions being inadequately evaluated.

Previously, we have explored the impact of a completely-remote multimodal digital intervention (DI) for other MSK conditions [36,37,38,39,40,41], as well as for rehabilitation after hip arthroplasty [42]. This study aimed to assess the clinical outcomes and engagement of this DI in patients with chronic hip pain. We hypothesized that patients would report improvement in outcomes (functionality, pain, mental health, and productivity) after the DI.

## 2. Materials and Methods

### 2.1. Study Design

This interventional, decentralized, single-arm study was performed on individuals with chronic hip pain who underwent a completely remote multimodal DI. The study was conducted in accordance with the Declaration of Helsinki. The trial was approved by the New England Institutional Review Board (number 120190313) and prospectively registered on ClinicalTrials.gov (NCT04092946, 17 September 2019). The DI was delivered between 20 June 2020 and 8 February 2022.

### 2.2. Participants

Individuals participating in health plans of employers from 47 states in the US, older than 18 years of age with self-reported hip pain complaints lasting longer than 3 months were invited to apply on an assigned website for Sword Health’s DI. Exclusion criteria included: (1) A health condition (e.g., cardiac, respiratory) incompatible with at least 20 min of light to moderate exercise, (2) receiving treatment for active cancer, and (3) reporting rapidly progressive loss of strength and/or numbness in the arms/legs or unexplained change in bowel or urinary function in the previous 2 weeks.

### 2.3. Intervention

**Figure 1:**
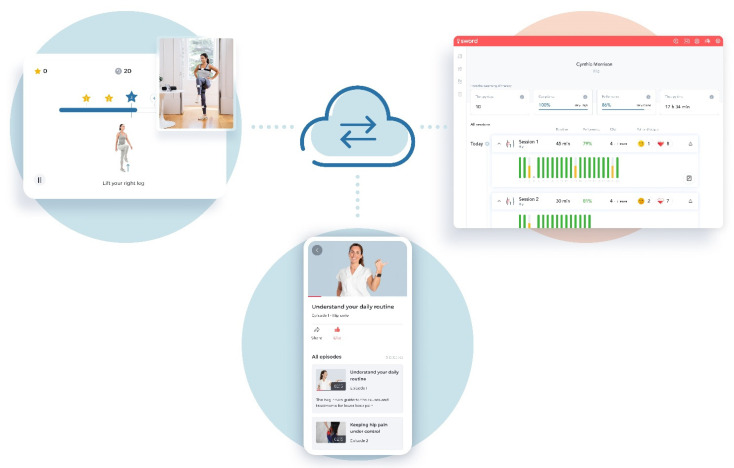
Schematic representation of the intervention. The top-left figure depicts a patient performing a session with motion trackers placed on the body transmitting the sensor data to the cloud. The top-right figure shows the web-based portal with the data of a patient’s sessions which can be assessed by the PT, who can adjust the program as needed. The bottom-center figure shows examples of educational articles on the dedicated smartphone app educating patients about their condition and pain self-management.

The intervention was a home-based DI comprised of exercise, education, and cognitive behavioral therapy (CBT), under the monitoring of a dedicated physical therapist (PT). Sword’s digital therapist is an FDA-listed, class II medical device, which is composed of three interconnected components: (1) a motion capture system composed of proprietary wearable motion-tracking sensors (inertial motion units capable of 9-axis movement capture), (2) a mobile App that comes pre-installed on an Android-based tablet, which guides the patient in each exercise session, and (3) a Web-based portal that allows the PT to define and edit the program (add/remove/edit exercises, difficulty levels, and goals) and gathers all the information from every session enabling remote monitoring (through analysis of patient performance, including correct and incorrect movements as well as the range of motion). Exercise sessions were performed using sensors placed over the patient’s body: chest, anterior surface of the hip, and over the anterior tibial crest (Figure 1). Before each exercise, patients were presented with a real-life video and audio explanation of that exercise. An execution interface was subsequently shown, with real-time audio and video feedback based on data captured by the motion trackers. For each correct repetition, the patient earned from 1 to 5 stars, depending on the range of motion of that specific movement in comparison to the target. The recommended exercise frequency was at least 3 sessions per week during a 12-weeks intervention (although early discharge was possible depending on the condition and PT assessment).

The educational (specifically targeting chronic hip pain) and CBT components were delivered through educational articles and interactive modules, available through a dedicated smartphone app. The content targeted pain reconceptualization, fear-avoidance, advice on the adoption of healthy lifestyles, the importance of exercise, activity pacing/modification, and self-management, according to the latest clinical guidelines and research [9,12]. The CBT program was based on third-generation techniques—mindfulness, acceptance, commitment therapy, and empathy-focused therapy. This program was created by an interdisciplinary team that included psychiatrists and psychologists and consisted of self-guided interactive modules, including pre-recorded audio sessions as well as habit releaser tasks compounded into different modules of empowering self-management tools.

Communication between the PT and patient was accomplished either through a secure chat within the aforementioned smartphone app or video call. Frequent communication was warranted to facilitate therapeutic alliance in a digital format and facilitate monitoring of potential adverse events.

### 2.4. Outcomes Measures

Outcomes were collected at baseline, 4-, 8-, and 12-weeks. Mean changes were estimated between baseline and 12 weeks for each outcome. 

The primary outcome was the Hip Disability and Osteoarthritis Outcome Score (HOOS), a 40-item questionnaire validated to assess symptoms and functional status related to hip conditions (either with or without osteoarthritis), that consisted of 5 sub-scales: Pain, Function, Quality of Life (QoL), Sport, and Symptom [43]. Each question was answered through a 5-point Likert scale, and a score is calculated per sub-scale (range 0–100), with higher scores reflecting better health status. 

The secondary outcomes included:

- Pain intensity assessed by the 11-point Numerical Pain Rating Score (NPRS) through the question “Please rate your average pain over the last 7 days: 0 (no pain at all) to 10 (worst pain imaginable)” [44].
- Self-reported surgery intent assessed by the question “How likely are you to seek surgery to address your condition in the next 12 months?” (range 0 (not likely)–100 (extremely likely)).
- Generalized Anxiety Disorder 7-item scale (GAD-7) (scores 0–21) was applied to assess anxiety severity in clinical practice and research, and was chosen due to strong validity and reliability: Cronbach’s alpha = 0.92 (excellent), ICC = 0.83 (excellent) [45,46]. Patient Health 9-item questionnaire (PHQ-9) (scores 0–27) was chosen for its strong scale validity (area under the curve in diagnosing major depression = 0.95), and reliability (Cronbach’s alpha = 0.89 (excellent)) as a brief measure of depression severity. Both scales evaluated symptomatology in the past two weeks. A cut-off threshold of ≥5 indicates at least mild anxiety/depression, respectively [45,47].
- Work productivity assessed by Work Productivity and Activity Impairment questionnaire for general health (WPAI), comprising the sub-scores: WPAI overall (combines presenteeism and absenteeism), WPAI work (presenteeism), WPAI time (absenteeism) in employed participants, and WPAI activities (non-work related activities impairment) in the entire cohort (range 0–100%, higher scores depicting greater impairment) [48].
- Patient engagement assessed by completion of the program (completion rate), cumulative time dedicated to exercise, number of completed exercise sessions, and number of sessions per week.
- Satisfaction by the question: “On a scale from 0 to 10, how likely is it that you would recommend this intervention to a friend or neighbor?”.

### 2.5. Safety and Adverse Events

Patients were advised to report any adverse events (e.g., worsening of symptomatology, new symptoms, or other events that could interfere with the condition or the execution of the program) to the dedicated PT through the available communication channels for further assessment. Additionally, pain and fatigue levels during the exercise sessions (assessed by NRS, range 0–10) were collected at the end of each session.

### 2.6. Data Availability

All relevant data are included in the article or available as supplementary material. De-identified data and analysis codes may be provided upon reasonable request to the corresponding author.

### 2.7. Statistical Analyses

Participants’ demographic characteristics (age, body mass index (BMI), sex, laterality, and employment status) and engagement measures were reported through descriptive statistics. Continuous variables were reported as mean (standard deviation—SD) and categorical variables as frequencies (%). Differences in baseline characteristics were explored between completers and non-completers (participants who were excluded or dropped out after the program started). Participants were considered completers if they were compliant with the intervention, independently of not completing a given reassessment survey, while those who did not perform exercise sessions for 28 consecutive days were considered dropouts.

Chi-square tests were used for categorical variables and independent samples t-tests for continuous variables. To assess the decrease in absenteeism between the start and the end of the program, the one-sided two-proportion Z-test was used.

Latent growth curve analysis (LGCA) was used to estimate clinical outcome trajectories across the intervention based on the individual trajectories and considering time as a continuous variable. This methodology belongs to the same family of linear mixed-effects modeling but is estimated as a structural equation model [49] (see Supplementary Figure S1), which considers repeated measures on the same individual to be correlated. LGCA includes measures of model fit and full information maximum likelihood (FIML) to deal with missing data [50,51]. FIML considers all available data at each time point from all participants. Previous research supports the superiority of FIML compared to other imputation methods [50,51].

All analyses were conducted as intent-to-treat. Results were also estimated after filtering cases for clinically relevant scores at baseline: Surgery Intent and WPAI outcomes >0; GAD-7 and PHQ-9 ≥ 5 points [47]. A conditional analysis was performed to assess the impact of age, sex, and BMI covariates on outcome changes. Models were adjusted for the aforementioned covariates, fitted as random effects, allowing variation between individuals. The impact of engagement on outcomes was evaluated using cumulative time dedicated to exercise sessions as a time-invariant covariate in the model. All models were estimated with a robust sandwich estimator for standard errors.

Taking into consideration that the available minimal clinically important changes (MCIC) scores in the literature for HOOS were solely derived from patients undergoing surgical procedures, response to treatment was evaluated using pain intensity as outcome, similarly to Dahlberg and colleagues [52]. An MCIC of 30% was applied to estimate responders to treatment as recommended by IMMPACT guidelines (referring to meaningful important improvements in subjects with chronic pain) [53]. A logistic regression analysis was conducted to assess the association of baseline outcomes with the odds of being a treatment responder.

Significance levels were considered as *p* < 0.05 in all analyses. LGCA was coded using R (version 1.4.1717) and all other analyses using SPSS (version 17.0, SPSS Inc., Chicago, IL, USA).

## 3. Results

### 3.1. Participants

**Figure 2:**
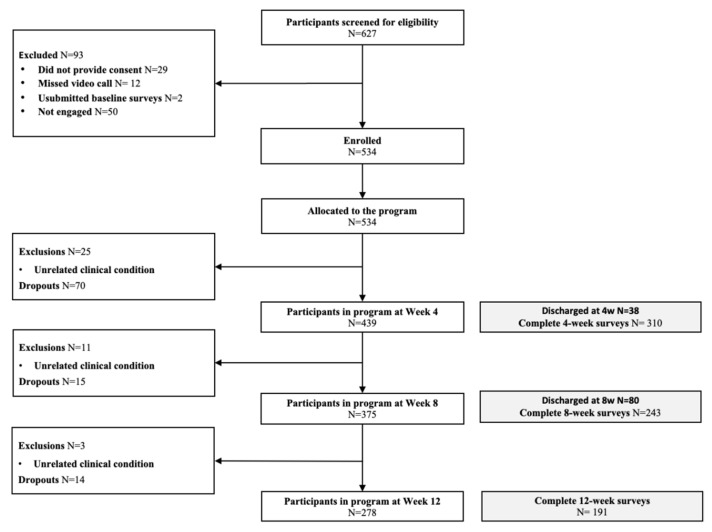
Study flow chart following CONSORT guidelines.

From 617 screened participants, 29 did not provide consent, 12 missed the video call, 2 did not submit the baseline survey, and 50 did not start (Figure 2). In total, 534 subjects started the program, and 396 completed the intervention (74.2% completion rate). No serious adverse events [54] were reported during the study. On average, participants were middle-aged (mean 50.2, SD 11.3), overweight (mean BMI 29.1, SD 6.4), and the majority were females (68.0%) (Table 1). No differences were found between completers (N = 396) and non-completers (N = 138) other than younger age (*p* = 0.002) and higher baseline levels of depressive symptoms (*p* = 0.009) in non-completers (Supplementary Table S1).

### 3.2. Clinical Outcomes

#### 3.2.1. Primary Outcome

##### HOOS

**Figure 3:**
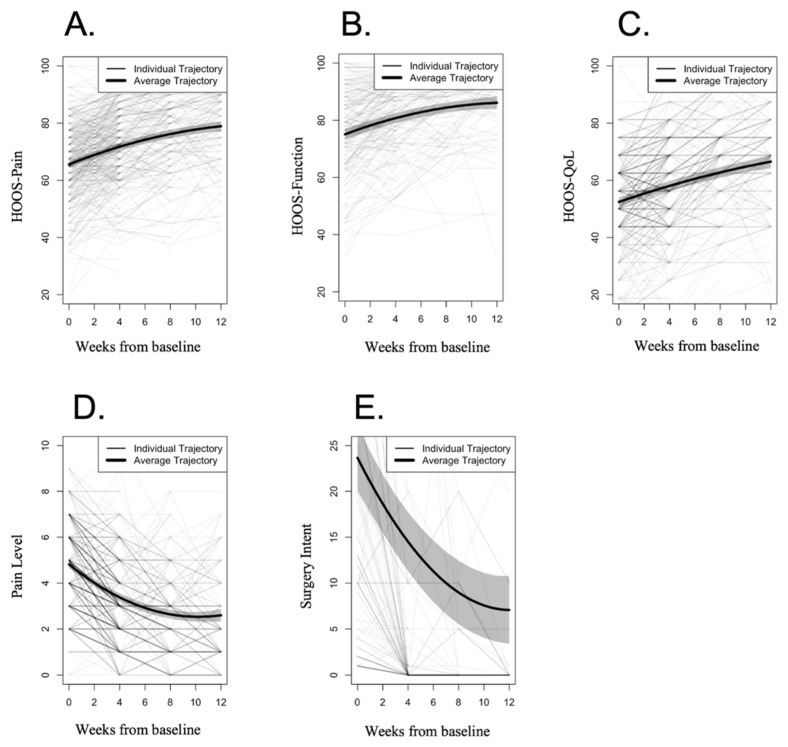
Longitudinal trajectories across time. Primary Outcomes: (**A**) HOOS-Pain; (**B**) HOOS-Function; (**C**) HOOS-QoL; (**D**) Pain Level; (**E**) Surgery Intent. Lighter lines represent individual trajectories (with darker lines indicating overlap of trajectories), while average trajectories calculated through LGCA are depicted in bold lines, with shadows representing 95% confidence intervals.

At baseline HOOS-QoL was the domain with the worst score (mean 52.44, SD 16.20). Significant improvements were found in all HOOS sub-scales (Table 2 and Figure 3): 13.32 points (95%CI 11.67; 14.97) in HOOS-Pain (20.3% improvement), 10.43 points (95%CI 8.20; 12.67) in HOOS-Symptoms (15.3% improvement), 11.01 points in HOOS-Function (95%CI 8.61; 13.41, 14.7% improvement), and 14.08 points (26.8%, 95%CI 12.03; 16.12) in HOOS-QoL (26.8% improvement). 

Those with higher BMI at baseline reported worse scores in all the HOOS sub-scales. Additionally, these participants had slower improvement rates in HOOS Function and Symptoms sub-scales (Supplementary Table S2).

#### 3.2.2. Secondary Outcomes

##### Pain

A significant reduction of 2.22 points (95%CI 1.93; 2.50; 46%) (Table 2 and Figure 3) was observed for pain at intervention end (*p* < 0.001, Supplementary Table S3). Among completers, 66.8% of participants surpassed the MCIC of 30% reduction in pain [53].

Older participants reported higher pain intensity at baseline (*p* < 0.001). However, they experienced a steeper reduction in pain across the intervention (Supplementary Table S2).

Pain reduction was correlated with the change in the different HOOS sub-scales (*p* < 0.001 for all) (-Pain: r(183) = −0.556; -Function: r(99) = −0.404); -QoL r(183) = −0.357); -Sport: r(99) = −0.432; -Symptoms: r(99) = −0.424), suggesting that higher pain reductions were associated with greater improvements in all HOOS sub-scales.

##### Surgery Intent

Among those reporting willingness to address their condition with surgery in the future, a decrease of 70.1% (16.59 points, 95%CI 8.61) was found after the intervention (Table 2 and Figure 3). Females reported a significantly greater decrease in their intention to undergo surgery after the DCP compared to males (Supplementary Tables S2). The overall change in surgery intent was correlated with improvements in pain (r(191) = 0.155, *p* = 0.033), HOOS-Function (r(99) = −0.284, *p* = 0.004) and HOOS-Symptoms (r(99) = −0.224, *p* = 0.026).

##### Mental Health

When filtering for cases with at least mild anxiety and depression (defined by scores ≥5 in both GAD-7 and PHQ-9) at baseline, patients scored on average 9.19 points in GAD-7 and 9.86 points in PHQ-9. These results are close to a moderate level of depression and anxiety (≥10 [47]) (Table 2). Significant improvements were observed in both mental health outcomes within those cohorts (*p* < 0.001), with a mean change of 54.1% in GAD-7 (4.97 points, 95%CI: 3.79; 6.15) and 54.6% in PHQ-9 (5.38 points, 95%CI: 4.23; 6.54). Anxiety improvements were correlated with reductions in pain (r(191) = 0.265, *p* < 0.001), as well as improvements in the following HOOS sub-scales: -Pain (r(183) = −0.183, *p* = 0.013); -QoL (r(183) = −0.207, *p* = 0.005) and -Symptoms (r(99) = −0.230, *p* = 0.022).

##### Work productivity

Absenteeism was very low at baseline, with only 10.5% of participants (45/430) reporting some degree of condition-related absenteeism, compromising the analysis by LGC. Nevertheless, a reduction to 3.9% (6/152) was observed by the study end (*p* = 0.007). Presenteeism, more frequently reported at baseline, was decreased by 59.3% (17.46, 95%CI 14.18; 20.74, *p* < 0.001) at the program end. Overall, a 60.5% improvement (18.25, 95%CI 14.92; 21.58, *p* < 0.001) was observed in work productivity impairment (WPAI Overall). This improvement was correlated with the change in surgery intent (r(135) = 0.173, *p* = 0.045) and improvements in HOOS -Pain (r(131) = −0.285, *p* = 0.001), -Function (r(71) = −0.302, *p* = 0.010), and -QoL (r(131) = −0.285, *p* = 0.001).

Non-work-related activities impairment was improved by 52.0% (WPAI Activity 18.56, 95%CI 15.11; 22.01, *p* < 0.001).

Covariates exerted no influence on recovery trajectories in any WPAI sub-scale (Supplementary Table S2).

WPAI Activity improvement was correlated with the improvement of all clinical outcomes: pain (r(191) = 0.367, *p* < 0.001), surgery intent (r(191) = 0.158, *p* = 0.029), GAD-7 (r(191) = 0.233, *p* = 0.001), PHQ-9 (r(191) = 0.189, *p* = 0.009) and all the HOOs sub-scales (-Pain: r(183) = −0.305, *p* < 0.001); -Function: r(99) = −0.440, *p* < 0.001); -QoL r(183) = −0.344, *p* < 0.001); -Sport: r(99) = −0.278, *p* = 0.005); and -Symptoms: r(99) = −0.287, *p* = 0.004).

##### Engagement and Usability-related Outcomes

Considering all enrolled participants (i.e., including dropouts and exclusions), an average of 2.9 (SD 1.1) sessions per week was performed, with completers performing 3.1 (SD 1.0) sessions per week. Participants performed an average of 30.4 (SD 27.8) exercise sessions, with completers performing an average of 37.0 sessions (SD 18.7), corresponding to 521.2 (SD 254.5) minutes dedicated to exercise treatment times. The influence of exercise duration on outcome changes was assessed by estimating the difference in average trajectories per additional hour performed above the average (Supplementary Table S4). Results showed that increased time spent exercising was significantly associated with greater improvements in HOOS QoL (*p* = 0.009), WPAI Overall (*p* = 0.049), WPAI Work (*p* = 0.043) and surgery intent (*p* = 0.048).

Regarding the psychoeducational component, participants read on average 5.3 (SD 7.5) articles. Patients communicated with their PT through the built-in app chat in an average of 11.3 (SD 13.3) days throughout the DI. Satisfaction with the program was high, with an overall mean score of 8.7/10 (SD 1.6) reported by patients.

## 4. Discussion

### 4.1. Main Findings

This study showed very high patient engagement, satisfaction (8.7/10, SD 1.6) and completion rate (74.2%) with a completely remote DI. Significant improvements were observed in all studied primary and secondary outcomes. The HOOS sub-scales were improved by 20.3% for HOOS-Pain, 14.7% for HOOS-Function, 26.8% for HOOS-QoL, 20.7% for HOSS-Sport, and 15.3% for HOOS-Symptoms. Correlations were found between changes in all HOOS sub-scales and all other secondary outcomes. Pain was significantly reduced by 2.22 points, with 66.8% of participants reporting ≥30% reduction, the cut-off recommended by IMMPACT to define a clinically meaningful response. Marked improvements were found in mental health (54.1% in anxiety and 54.6% in depression) and work productivity domains (60.5% in overall productivity).

### 4.2. Comparison with the Literature

Digital interventions have only recently been applied for chronic hip conditions. These have leveraged the knowledge gathered after in-person care [55,56,57] to create remote programs combining both exercise and education [26,52,58]. The DI herein presented follows a biopsychosocial framework, encompassing CBT, education, and exercise, which seemed to be well accepted by patients, considering the high completion rate, engagement, and satisfaction with the program. The completion rate of 74.2% in this real-world context study was within the range of prior published studies evaluating either on in-person [55,56] or telerehabilitation interventions [26,52]. Contrary to most studies [26,58], engagement was assessed by objective metrics, showing almost complete adherence as most participants complied with the recommended exercise session frequency. This high commitment might result from important features included in the DI, previously identified in the literature to enhance patient adherence, such as communication with the assigned PT, accountability through continuous monitoring, gamification, and real-time biofeedback [25,59]. Furthermore, high engagement was associated with greater improvements in several outcomes, reinforcing the importance of stimulating patient engagement in rehabilitation.

The significant improvements in HOOS outcomes were similar or greater than those previously reported after in-person interventions (independent of the sub-scale) [55,56,57]. Skou et al. [57] reported the outcomes observed in a national-registry study focused on hip osteoarthritis management through exercise and education. Baseline values in the HOOS-QoL sub-scale were similar to those found in the present study, but outcomes both at three months (10.1% change) and long-term follow-up (12 months, 20.2% change) were below the improvement observed herein (26.8%). Interestingly, we also found that improvement in HOOS-QoL was augmented with higher patient engagement.

Most DI report improvements that vary significantly depending on the HOOS sub-scale and on the condition’s severity [32,34]. Many studies report aggregated results from cohorts with mixed conditions (hip or knee pain), which makes direct comparisons challenging [31,35,60]. In a randomized-controlled trial (RCT) that compared usual physical therapy to exercise and education delivered in a blended format (in-person and remote), Kloek et al. [34] reported no differences between groups. HOOS improvement in the present study was greater than that reported at three months and similar to that observed at long-term reassessment, supporting this type of care delivery system. Further, HOOS improvements were correlated with the reductions observed in each secondary outcome, consistent with bidirectional interactions within a biopsychosocial framework [61,62].

Regarding pain intensity, we observed an average absolute change of 2.22 points, corresponding to a 66.8% of responders rate (MCIC of 30%). This percentage of responders is similar to that reported by Dahlberg and colleagues [52] (67–72%), who applied a less stringent cut-off (20%). Additionally, the average pain improvement described herein was greater than those found in other in-person [21,63] and digital rehabilitation studies (15–34%) [26,52,58].

Among interventions focused on exercise and education, in-person studies [21,63] have reported pain improvements below the herein described. Wide-reaching national initiatives delivering the aforementioned interventions digitally [26,52,58] have also reported pain improvements in a range below that observed herein. Bennell et al. [58] reported an RCT focused on the importance of adding behavioral change (pain coping skills) training to exercise and education in a blended intervention. The changes observed at the program end (eight weeks) (16–18%) were below the herein reported, although at the six-months follow-up (35–48.1%) similar improvements were reported to those found in our 12-weeks final assessment.

Current literature indicates that hip conditions are strongly associated with the patient’s levels of anxiety and depression [4,5]. Significant improvement in these two domains was observed after the DI, within a range (37–42%) greater than that previously reported both after in-person rehabilitation (28% to 29%) and telerehabilitation (6.3% to 35%) [31,32]. When focusing on participants with at least mild anxiety and depression symptomatology at baseline, even greater improvements were observed (54.1% in GAD-7 and 54.6% in PHQ-9), further supporting the use of this program in this challenging population. Chronic MSK conditions are intimately associated with work productivity impairment [64,65], with few studies examining the impact of conservative treatment on this domain. Absenteeism was not frequently observed at baseline but recovered to similar levels as those previously reported by Jonsson et al. [21] in patients with hip osteoarthritis who underwent a self-management program: sick leave decreased from 12% to 5%. Presenteeism and non-work-related activity impairment were more prevalent, in accordance with literature for chronic hip pain [65]. Nevertheless, significant improvements in presenteeism (59.3%) and in non-work-related activity impairment (52%) were observed. The recovery in productivity was commensurate with the improvements in function, pain, and mental health, reinforcing the importance of tackling different domains simultaneously when addressing hip pain. Overall, the DI positively impacted patients as higher engagement was associated with greater improvement in work productivity.

Contrary to recommendations [8,9,10,11,12], surgical management is still highly prevalent and often employed before adequate conservative approaches [13]. A patient’s willingness to undergo surgery is the most important impetus for total hip replacement [66]. However, most patients are unaware of the available treatment options [67], which sometimes leads to unnecessary surgery and concomitant risks and costs. Herein, we showed a significant reduction (70.1%) in surgery intent at the program end, which increased with increasing levels of engagement. This result is supported by previous observational studies highlighting the role of exercise-based approaches in changing patients’ attitudes towards surgery, either through in-person [68,69] or telerehabilitation [33,70].

In all, the outcomes observed herein were similar or greater than the reported in the literature, which likely reflects a variety of different factors, including the particular components of the intervention. The accessibility and convenience provided by the remote nature of this DI, the real-time feedback, the gamification of exercises, the accountability, and multiple communication channels [25,59] each might have contributed to the high engagement observed, which in turn contributed to the favorable outcomes. As recommended by previous clinical practice guidelines [9,12], our intervention includes a CBT component. Although the present study does not allow for inferences, one can speculate that the multimodal approach provided added value. 

### 4.3. Strengths and Limitations

The main strengths of the study include: (i) the large sample size comprised of various chronic hip conditions from geographically diverse states evaluated under current clinical practice, (ii) the novelty of the intervention (structured within a biopsychosocial framework), offered as a completely remote program accompanied by an assigned PT and real-time biofeedback. Additionally, the chosen outcome domains are consistent with International Consortium for Health Outcomes Measurement (ICHOM) standards for hip conditions [71], facilitating comparisons with future studies. Moreover, DI engagement was objectively measured, thereby minimizing social desirability response bias.

The major limitation is the lack of a control group. Considering the real-world context of the study, the most obvious control group would be “wait-listed patients”, which would not simulate clinical practice and may not be ethical. Notwithstanding, taken together, the results reported herein on engagement and observed outcomes are highly encouraging. Other limitations include failure to stratify the impact of each DI component and the lack of long-term follow-up to evaluate the durability of results.

## 5. Conclusions

We observed significant improvements in all clinical outcomes after this DI, which favorably compares with previously published results for in-person or telerehabilitation programs. Greater improvement in HOOS-QoL, surgery intent, and work productivity were attained in more engaged individuals, highlighting the importance of patient adherence. These auspicious findings reinforce the utility of a completely remote DI in the management of chronic hip pain. Future research may provide further insights on the effectiveness and efficiency of digital interventions compared with in-person PT or other digital programs and incorporate long-term follow-up.

## Figures and Tables

**Table 1:** Baseline characteristics of study participants (N = 534).

Characteristic	Entire Cohort (N = 534)
Age (years), mean (SD)	50.2 (11.3)
Age categories (years), N (%):	
<25	3 (0.6)
25–40	122 (22.8)
40–60	292 (54.7)
>60	117 (21.9)
Sex, N (%)	
Female	363 (68.0)
Male	171 (32.0)
BMI, mean (SD) ^a^	29.1 (6.4)
BMI categories, N (%) ^a^:	
Underweight (<18.5)	2 (0.4)
Normal (18.5–25)	147 (27.7)
Overweight (25–30)	188 (35.4)
Obese (30–40)	159 (29.9)
Morbidly obese (>40)	35 (6.6)
Laterality	
Left	150 (28.1)
Right	185 (34.6)
Both	199 (37.3)
Employment status, N (%):	
Employed (part-time or full-time)	480 (89.9)
Unemployed (not working or retired)	54 (10.1)
Hip pain-related condition, N (%):	
Hip Osteoarthritis	106 (19.9%)
Others ^b^	428 (80.1%)
Psychopathology comorbidity	
GAD-7 ≥ 5	135 (25.3%)
GAD-7 ≥ 10	46 (8.6%)
PHQ-9 ≥ 5	102 (19.1%)
PHQ-9 ≥ 10	34 (6.4%)

**Abbreviations:** BMI, body mass index. ^a^: 3 missing values; ^b^: other conditions include non-specific pain, bursitis, femoroacetabular syndrome, sprain/strain, gluteal tendinopathy, etc.

**Table 2:** Outcome changes between baseline and end-of-program: Intent-to-treat approach (unconditional model).

Outcome, Mean (95%CI)	*n*	Baseline	End-of-Program	Mean Change	% Change
HOOS-Pain	515	65.59 (64.33; 66.84)	78.91 (77.17; 80.65)	13.32 (11.67; 14.97)	20.3%
HOOS-Function	251	75.08 (73.17; 77.00)	86.09 (83.89; 88.30)	11.01 (8.61; 13.41)	14.7%
HOOS-Qol	515	52.44 (50.86; 54.02)	66.52 (64.22; 68.81)	14.08 (12.03; 16.12)	26.8%
HOOS-Sport	251	65.37 (62.84; 67.90)	78.92 (76.20; 81.63)	13.55 (10.76; 16.33)	20.7%
HOOS-Symptoms	251	68.18 (66.25; 70.12)	78.62 (76.38; 80.85)	10.43 (8.20; 12.67)	15.3%
Pain Level	534	4.82 (4.65; 4.98)	2.60 (2.33; 2.87)	2.22 (1.93; 2.50)	46.0%
Surgery Intent > 0	201	23.67 (20.10; 27.23)	7.07 (3.38; 10.77)	16.59 (12.92; 20.27)	70.1%
Surgery Intent	534	8.84 (7.14; 10.54)	3.16 (1.67; 4.65)	5.68 (4.01; 7.34)	64.3%
GAD-7 ≥ 5	135	9.19 (8.43; 9.94)	4.22 (3.07; 5.36)	4.97 (3.79; 6.15)	54.1%
GAD-7	534	3.05 (2.68; 3.42)	1.92 (1.51; 2.33)	1.13 (0.68; 1.57)	36.9%
PHQ-9 ≥ 5	102	9.86 (8.85; 10.87)	4.48 (3.27; 5.46)	5.38 (4.23; 6.54)	54.6%
PHQ-9	534	2.66 (2.29; 3.03)	1.55 (1.22; 1.87)	1.16 (0.75; 1.49)	41.9%
WPAI Overall > 0	224	30.18 (27.37; 33.00)	11.94 (8.63; 15.24)	18.25 (14.92; 21.58)	60.5%
WPAI Overall	430	15.82 (13.80; 17.84)	9.05 (6.35; 11.75)	6.77 (3.89; 9.66)	42.8%
WPAI Work > 0	218	29.43 (26.72; 32.14)	11.97 (8.72; 15.22)	17.46 (14.18; 20.74)	59.3%
WPAI Work	430	14.91 (13.00; 16.81)	9.05 (6.90; 11.47)	5.86 (2.93; 8.80)	39.3%
WPAI Activity > 0	390	35.70 (33.41; 37.98)	17.14 (13.75; 20.52)	18.56 (15.11; 22.01)	52.0%
WPAI Activity	534	26.07 (23.94; 28.20)	14.68 (11.88; 17.47)	11.39 (8.40; 14.38)	43.7%

**Abbreviations:** HOOS, Hip Disability and Osteoarthritis Outcome Score; GAD-7, Generalized Anxiety Disorder 7-item scale; PHQ-9, Patient Health 9-item questionnaire; WPAI, Work Productivity and Activity Impairment Questionnaire.

## Data Availability

The data presented in this study are available on request from the corresponding author. The data are not publicly available due to privacy restrictions.

## Supplementary Materials

The following supporting information can be downloaded at: www.mdpi.com/article/10.3390/healthcare10081595/s1, Figure S1: Example path diagram for the LGC models used in the current study; Table S1: Baseline characteristics of completers vs non-completers; Table S2: Intent-to-treat Conditional Latent Growth Curve Model, with age, sex and body mass index as covariates, Table S3: Latent Growth Curve analysis: intent-to-treat [72,73], Table S4: Effect of cumulative training time on the slopes of recovery trajectories for the different outcome variables.
