# Supplementary material for: Digital Care Programs for Chronic Hip Pain: A Prospective Longitudinal Cohort Study
Source: Healthcare (Basel). 2022 Aug 22;10(8):1595. doi: 10.3390/healthcare10081595 (PMC9408636; doi:10.3390/healthcare10081595)
Supplement: Supplementary file 1 [file healthcare-10-01595-s001.zip › healthcare-1843912-supplementary.pdf]

Supplementary Materials

Digital care programs for chronic hip pain: a prospective longitudinal cohort study

Dora Janela, Fabíola Costa, Anabela C. Areias, Maria Molinos, Robert G. Moulder, Jorge Lains, Virgílio Bento, Justin K Scheer, Vijay Yanamadala, Steven P. Cohen, Fernando Dias Correia

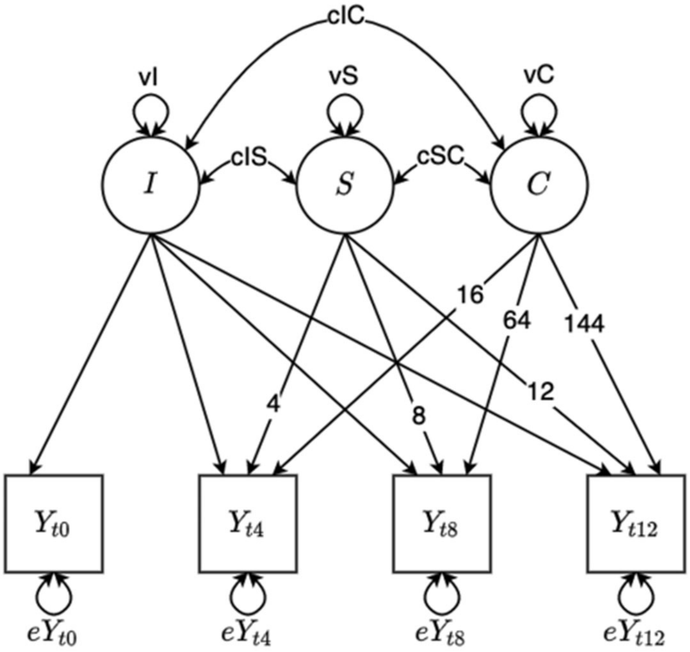

Supplementary Figure S1. Example path diagram for the LGC models used in the current study. LGCs are a form of structural equation model for modeling longitudinal processes. Squares represent outcome variables at baseline, 4 weeks, 8 weeks, and 12 weeks. Circles represent average latent intercept (I), slope (S), and curvature (C) components across all participants. Single-headed arrows represent fixed loadings relating the outcome variables to the latent components. Double-headed arrows represent either variance, covariance, or error variance parameters: variance of the intercept (vI), slope (vS) and curvature (vC); covariance between intercept and slope (cIS), slope and curvature (cSC), and intercept and curvature (cIC); error variance across measurement time points - at baseline (eY_{t0}), 4 (eY_{t4}), 8 (eY_{t8}) and 12 (eY_{t12}) weeks.

Latent growth curve (LGC) models take the form:

$$Y_{ij} = I + St_i + Ct_i + u_{Ij} + u_{sj}t_i + u_{cj}t_i + e_{ij}$$

where *Y* is the outcome score for person, *j*, at time, *i*, t_i is a vector of time points representing [0, 4, 8, 12] weeks, *I* is an intercept term representing the average value at *t=0* for all participants, *S* is a slope term representing the

average linear change of Y_{ij} over time for all participants, C is a curvature term representing the quadratic change over time of Y_{ij} for all participants, u_{ij} is a random intercept term representing individual participant variation in I, u_{sj} is a random slope term representing individual participant variation in S, u_{cj} is a random curvature term representing individual participant variation in C, and e_{ij} is an error term (see Supplementary Figure 1).

Supplementary Table S1. Baseline characteristics of completers vs non-completers.

**Characteristic**	**Completers (N=396)**	**Non-completers (N=138)**	**p**
Age (years), mean (SD)	50.7 (11.2)	48.7 (11.4)	**.002**
Age categories, N (%):			.090
<25	3 (0.8)	0 (0.0)	
25-40	83 (21.0)	39 (28.3)	
40-60	215 (54.3)	77 (55.8)	
> 60	95 (24.0)	22 (15.9)	
Sex, N (%):			.801
Female	268 (67.7)	95 (68.8)	
Male	128 (32.3)	43 (31.2)	
BMI, mean (SD)[a]	27.6 (5.7)	28.1 (5.3)[a]	.416
BMI categories, N (%)[a]:			.150
Underweight (<18.5)	0 (0.0)	2 (0.5)	
Normal (18.5-25)	32 (23.4)	115 (29.2)	
Overweight (25-30)	43 (31.4)	145 (36.8)	
Obese (30-40)	50 (36.5)	109 (27.7)	
Morbidly obese (>40)	12 (8.8)	23 (5.8)	
Laterality, N (%):			.595
right	141 (35.6)	44 (31.9)	
left	107 (27.0)	43 (31.1)	
both	148 (37.4)	51 (37.0)	
Employment status, N (%):			.732
Employed (part-time or full-time)	357 (90.2)	123 (89.1)	
Unemployed	39 (9.8)	15 (10.9)	
Hip pain-related condition, N (%):			.400
Hip Osteoarthritis	82 (20.7)	24 (17.4)	
Others[d]	314 (79.3)	114 (82.6)	
Psychopathology comorbidity, N (%):			
GAD-7≥ 5	94 (23.7%)	41 (29.7%)	.164
GAD-7≥ 10	30 (7.6%)	16 (11.6%)	.147
PHQ-9≥ 5	65 (16.4%)	37 (26.8%)	**.007**
PHQ-9≥ 10	14 (3.5%)	20 (14.5%)	**<.001**
Outcome measures, mean (SD):			
HOOS-Pain[b]	65.5 (14.3)	65.7 (15.6)	.848
HOOS-Function[c]	75.1 (14.2)	75.1 (16.3)	.987
HOOS-Qol[b]	52.5 (18.3)	52.5 (18.2)	.969
HOOS-Sport[c]	65.3 (20.8)	66.3 (22.1)	.749

HOOS-Symptoms[c]	68.7 (15.6)	66.7 (16.1)	.400
Pain Level	4.8 (1.9)	5.0 (1.9)	.160
Surgery Intent	8.9 (19.5)	8.9 (19.8)	.983
GAD-7	2.8 (3.9)	3.7 (5.4)	.099
PHQ-9	2.3 (3.6)	3.7 (5.9)	**.009**
WPAI Overall	15.5 (21.6)	18.1 (23.2)	.298
WPAI Work	14.6 (20.1)	17.2 (22.6)	.250
WPAI Time	2.3 (12.2)	3.6 (15.0)	.342
WPAI Activity	25.5 (24.2)	28.5 (27.5)	.257

Abbreviations: *BMI, Body mass index; HOOS, Hip Disability and Osteoarthritis Outcome Score; GAD-7, Generalized Anxiety Disorder 7-item scale; PHQ-9, Patient Health 9-item questionnaire; WPAI, Work Productivity and Activity Impairment Questionnaire. Missing data: a: N=3; b: N=19; c: N=283. d: other conditions include non-specific pain, bursitis, femoroacetabular syndrome, sprain/strain, gluteal tendinopathy, etc. Note: Significant p-values are presented in bold.*

Supplementary Table S2. Intent-to-treat Conditional Latent Growth Curve Model, with age, sex and body mass index as covariates.

	**Age**			**Female**			**BMI**		
Outcome	Intercept	Slope	Curve	Intercept	Slope	Curve	Intercept	Slope	Curve
Hoos-Pain	**-0.20**	0.02	0	**-4.15**	**1.00**	-0.05	**-0.39**	-0.02	0
	**(< .001)**	(0.262)	(0.422)	**(0.002)**	**(0.016)**	(0.116)	**(< .001)**	(0.568)	(0.401)
Hoos-Function	**-0.24**	0.02	0	**-5.44**	0.90	-0.03	**-0.63**	**0.14**	**-0.01**
	**(0.002)**	(0.338)	(0.712)	**(0.005)**	(0.080)	(0.523)	**(< .001)**	**(0.003)**	**(0.003)**
Hoos-Qol	-0.13	0.03	0	-0.73	-0.12	0.03	**-0.35**	0.03	0
	(0.066)	(0.193)	(0.184)	(0.681)	(0.803)	(0.436)	**(0.005)**	(0.470)	(0.223)
Hoos-Sport	-0.11	-0.02	0	-3.04	-0.35	0.06	**-0.48**	0.09	-0.01
	(0.307)	(0.568)	(0.476)	(0.254)	(0.655)	(0.300)	**(0.036)**	(0.286)	(0.170)
Hoos-Symptoms	-0.02	0.01	0	-3.87	0.30	0.01	**-0.46**	**0.13**	**-0.01**
	(0.808)	(0.698)	(0.920)	(0.062)	(0.603)	(0.839)	**(0.015)**	**(0.015)**	**(0.005)**
Pain Level	**0.03**	**-0.01**	**0**	**0.37**	-0.05	0	**0.04**	0	0
	**(< .001)**	**(0.012)**	**(0.016)**	**(0.032)**	(0.388)	(0.638)	**(0.001)**	(0.437)	(0.436)
Surgery Intent >0	0.03	0.02	0	-2.85	**-1.93**	**0.16**	0.51	0.05	0
	(0.892)	(0.610)	(0.658)	(0.447)	**(0.011)**	**(0.001)**	(0.071)	(0.406)	(0.310)
Surgery Intent	0.14	-0.01	0	-3.19	**-0.80**	**0.07**	**0.29**	0.01	0
	(0.071)	(0.461)	(0.995)	(0.102)	**(0.047)**	**(0.008)**	**(0.041)**	(0.677)	(0.616)
GAD-7 ≥ 5	**-0.07**	0.01	0	-0.08	-0.07	0.01	0.04	-0.01	0
	**(0.019)**	(0.484)	(0.310)	(0.931)	(0.816)	(0.696)	(0.544)	(0.684)	(0.543)
GAD-7	**-0.06**	0	0	0.62	-0.02	0	**0.07**	0	0
	**(< .001)**	(0.308)	(0.325)	(0.115)	(0.835)	(0.970)	**(0.036)**	(0.692)	(0.255)
PHQ-9 ≥ 5	-0.05	-0.02	0	-1.58	-0.19	0.02	0.10	-0.01	0
	(0.177)	(0.325)	(0.384)	(0.141)	(0.723)	(0.624)	(0.182)	(0.711)	(0.981)
PHQ-9	-0.03	0	0	-0.23	0.06	-0.01	**0.10**	0	0
	(0.062)	(0.429)	(0.433)	(0.588)	(0.561)	(0.414)	**(0.006)**	(0.639)	(0.535)
WPAI Overall >0	**-0.30**	-0.01	0	-1.74	-0.47	0	0.30	-0.1	0.01
	**(0.011)**	(0.851)	(0.620)	(0.579)	(0.665)	(0.957)	(0.179)	(0.228)	(0.340)
WPAI Overall	**-0.21**	0	0	0.02	-0.5	0.03	**0.41**	-0.03	0
	**(0.028)**	(0.975)	(0.574)	(0.992)	(0.456)	(0.544)	**(0.021)**	(0.703)	(0.696)

WPAI Work >0	**-0.23 (0.034)**	-0.03 (0.548)	0 (0.385)	-2.18 (0.479)	-0.75 (0.458)	0.03 (0.732)	**0.42 (0.040)**	-0.13 (0.080)	0.01 (0.146)
WPAI Work	**-0.17 (0.043)**	-0.01 (0.634)	0 (0.256)	-0.53 (0.800)	-0.65 (0.306)	0.06 (0.314)	**0.43 (0.011)**	-0.05 (0.418)	0 (0.380)
WPAI Activity >0	0.12 (0.192)	-0.05 (0.224)	0 (0.233)	2.32 (0.351)	-1.08 (0.259)	0.03 (0.675)	**0.41 (0.020)**	-0.11 (0.120)	0.01 (0.112)
WPAI Activity	0.04 (0.654)	-0.02 (0.464)	0 (0.384)	3.9 (0.088)	-1.28 (0.087)	0.08 (0.181)	**0.63 (< .001)**	-0.12 (0.055)	0.01 (0.071)

Abbreviations: *HOOS, Hip Disability and Osteoarthritis Outcome Score; GAD-7, Generalized Anxiety Disorder 7-item scale; PHQ-9, Patient Health 9-item questionnaire; WPAI, Work Productivity and Activity Impairment Questionnaire. Note: Significant p-values are presented in bold.*

Supplementary Table S3. Latent Growth Curve analysis: intent-to-treat.

		**Intercept**		**Slope**		**Curve**		**Fit**				
Outcome	N	Mean (SD)	p	Mean (SD)	p	Mean (SD)	p	Chi-sq (df)	p	RMSEA	CFI	SRMR
HOOS-Pain	515	65.59 (12.16)	**< .001**	1.75 (2.07)	**< .001**	-0.05 (0.15)	**0.001**	0.2 (1)	**0.656**	**0.000**	**1.00**	**0.004**
HOOS-Function	251	75.08 (14.84)	**< .001**	1.67 (2.01)	**< .001**	-0.06 (0.12)	**0.002**	0.48 (1)	**0.488**	**0.000**	**1.00**	**0.008**
HOOS-Qol	515	52.44 (16.20)	**< .001**	1.52 (2.22)	**< .001**	-0.03 (0.13)	0.152	4.18 (1)	0.041	**0.078**	**0.99**	**0.017**
HOOS-Sport	251	65.37 (20.16)	**< .001**	1.92 (3.65)	**< .001**	-0.07 (0.24)	**0.030**	0.56 (1)	**0.455**	**0.000**	**1.00**	**0.009**
HOOS-Symptoms	251	68.18 (15.08)	**< .001**	1.10 (2.77)	**< .001**	-0.02 (0.18)	0.407	0.96 (1)	**0.327**	**0.000**	**1.00**	**0.012**
Pain Level	534	4.82 (1.81)	**< .001**	-0.45 (0.39)	**< .001**	0.02 (0.03)	**< .001**	26.06 (1)	< .001	0.217	**0.91**	0.059
Surgery Intent >0	201	23.67 (25.8)	**< .001**	-2.74 (4.21)	**< .001**	0.11 (0.22)	**< .001**	6.63 (1)	0.010	0.167	**0.98**	**0.048**
Surgery Intent	534	8.84 (18.52)	**< .001**	-0.81 (2.93)	**< .001**	0.03 (0.16)	**0.009**	9.04 (1)	0.003	0.123	**0.99**	**0.043**
GAD-7 ≥ 5	135	9.19 (4.46)	**< .001**	-0.80 (0.99)	**< .001**	0.03 (0.05)	**0.009**	0.85 (1)	**0.357**	**0.000**	**1.00**	**0.036**
GAD-7	534	3.05 (4.26)	**< .001**	-0.13 (0.72)	**0.013**	0.00 (0.03)	0.484	5.53 (1)	0.019	0.092	**0.99**	**0.021**
PHQ-9 ≥ 5	102	9.86 (5.20)	**< .001**	-1.14 (1.49)	**< .001**	0.06 (0.09)	**0.001**	0.84 (1)	**0.360**	**0.000**	**1.00**	**0.040**
PHQ-9	534	2.66 (4.18)	**< .001**	-0.22 (0.76)	**< .001**	0.01 (0.03)	**0.007**	2.58 (1)	0.108	**0.054**	**1.00**	**0.017**
WPAI Overall >0	224	30.18 (10.09)	**< .001**	-2.66 (0.03)	**< .001**	0.10 (0.16)	0.053	11.52 (1)	< .001	0.217	**0.91**	0.065
WPAI Overall	430	15.82 (13.77)	**< .001**	-0.95 (0.03)	**0.01**	0.03 (0.13)	0.313	9.02 (1)	0.003	0.132	**0.96**	**0.044**
WPAI Work >0	218	29.43 (12.9)	**< .001**	-2.44 (0.03)	**< .001**	0.08 (0.11)	0.071	10.51 (1)	0.001	0.209	**0.93**	0.063
WPAI Work	430	14.91 (13.84)	**< .001**	-0.87 (0.03)	**0.011**	0.03 (0.14)	0.304	7.45 (1)	0.006	0.118	**0.97**	**0.040**
WPAI Activity >0	390	35.70 (17.03)	**< .001**	-3.42 (3.04)	**< .001**	0.16 (0.03)	**< .001**	5.15 (1)	0.023	0.103	**0.98**	**0.032**

WPAI Activity	534	26.07 (18.54)	**< .001**	-2.13 (2.47)	**< .001**	0.10 (0.03)	**0.002**	2.36 (1)	**0.124**	**0.050**	**1.00**	**0.018**

Abbreviations: *HOOS, Hip Disability and Osteoarthritis Outcome Score; GAD-7, Generalized Anxiety Disorder 7-item scale; PHQ-9, Patient Health 9-item questionnaire; WPAI, Work Productivity and Activity Impairment Questionnaire.*

Each outcome average trajectory is described through intercept, slope and curve. The intercept refers to the initial estimated value at baseline, the slope the linear outcome mean change per week and the curve the possible leveling effect towards the end of the program. As an example, the average baseline score on Pain was 4.82 (SD 1.81), with a significant average reduction of 0.45 (SD 0.39) points per week.

Model fit estimation was assessed through chi-squared test, confirmatory fit index (CFI), root mean square error of approximation (RMSEA), and standardized root mean square residual (SRMR), based on the following thresholds: CFI = close to .95; RMSEA = close to .06 and SRMR = close to .08 [72,73]. Note: Significant p-values are presented in bold.

Supplementary Table S4. Effect of cumulative training time on the slopes of recovery trajectories for the different outcome variables.

Outcome	Estimate	P
Hoos-Pain	0.000	0.933
Hoos-Function	0.000	0.974
Hoos-Qol	-0.011	**0.009**
Hoos-Sport	-0.004	0.517
Hoos-Symptoms	-0.006	0.152
Pain Level	-0.001	0.083
Surgery Intent >0	-0.012	**0.048**
Surgery Intent	-0.005	0.083
GAD-7 ≥ 5	-0.003	0.192
GAD-7	0.000	0.568
PHQ-9 ≥ 5	-0.002	0.456
PHQ-9	-0.001	0.911
WPAI Overall > 0	-0.020	**0.049**
WPAI Overall	-0.015	**0.002**
WPAI Work > 0	-0.018	**0.043**
WPAI Work	-0.015	**0.001**
WPAI Activity > 0	-0.010	0.138
WPAI Activity	-0.006	0.306

Abbreviations: *HOOS, Hip Disability and Osteoarthritis Outcome Score; GAD-7, Generalized Anxiety Disorder 7-item scale; PHQ-9, Patient Health 9-item questionnaire; WPAI, Work Productivity and Activity Impairment Questionnaire.*

A negative estimate refers to an end score lower than the average. E.g: for HOOS QoL, each hour performed above the average cumulative time dedicated to exercise (mean 421.1 minutes), would result in a final score of 4.632 points lower than the average at the end the program. Significant p-values are presented in bold.
